# Travel-associated COVID-19: a challenge for surveillance?

**DOI:** 10.2807/1560-7917.ES.2020.25.37.2001641

**Published:** 2020-09-17

**Authors:** Julien Beauté, Gianfranco Spiteri

**Affiliations:** 1 European Centre for Disease Prevention and Control (ECDC), Stockholm, Sweden

**Keywords:** COVID-19, surveillance, travel-associated infections, transients, migrants

The coronavirus disease (COVID-19) pandemic started in late 2019 in Wuhan, China before spreading globally in the following months, first in Asia and Europe, followed by the Americas, Africa and Oceania [1]. The substantial morbidity and mortality associated with the pandemic led countries to implement exceptional public health measures, including stay-at-home recommendations and orders. In many countries, observed epidemiological patterns were similar and initially imported cases were soon followed by community transmission [2,3]. In countries that managed to control the local epidemics, reporting of cases continued at lower levels, with a large proportion of imported cases in some [4]. In Europe, the proportion of imported cases increased in June and July 2020 compared with previous months, probably driven by testing that targeted travellers as travel restrictions were eased [5]. Cases reported as imported encompass a variety of situations that may challenge surveillance indicators.

## Surveillance of COVID-19 in Europe

Surveillance of COVID-19 in Europe started on 27 January 2020 when the European Centre for Disease Prevention and Control (ECDC) and the World Health Organization (WHO) Regional Office for Europe asked countries to report all cases meeting the WHO case definition [6]. The initial objectives were to describe the key epidemiological and clinical characteristics of COVID-19 cases detected in Europe, to inform country preparedness and to improve case detection and management. These objectives were revised in April 2020 and emphasis was put on monitoring the intensity, time trends and geographical spread of the severe acute respiratory coronavirus 2 (SARS-CoV-2) in the population [7]. To achieve these objectives, ECDC recommended that countries carry out comprehensive national surveillance to allow calculation of the notification rate per 100,000 population. This rate is an accurate indicator of the transmission intensity and provides a sound basis for monitoring trends over time, if the testing strategy remains unchanged. Since 25 June 2020, ECDC has produced weekly reports of 14-day notification rates of new COVID-19 cases at national and subnational levels for European Union/European Economic Area (EU/EEA) countries. The numbers of COVID-19 cases used for calculating the notification rates are collected by searching information on official websites (e.g. from public health authorities) or reported by EU/EEA countries through the Early Warning and Response System (EWRS). All cases have been included in the numerator of the notification rate, but it could be argued that imported cases distort the rate, which is supposed to reflect the intensity of community transmission in a given area.

Countries also report their cases to The European Surveillance System (TESSy)–which is mostly case based– with a set of variables including importation status (i.e. case travelled outside the reporting country in the 14 days before symptom onset) and geographical information (i.e. place of residence and place of infection). Provided that these variables have a good completeness, it is theoretically possible to distinguish locally acquired infections from those that were imported. However, it remains to be determined whether this distinction makes sense and if it does, how to best use this information to meet the surveillance objectives.

## Surveillance of travel-associated infections

There are a number of challenges related to both the definition and the classification of travel-associated infections.

An imported case is usually defined as any case with a travel history outside of the reporting country in a specified number of days before onset of symptoms, i.e. 14 days for COVID-19 surveillance in Europe. Since most Europeans travel domestically, a substantial proportion of travel-associated COVID-19 will not be captured [8]. This may be of importance considering the heterogeneity of SARS-CoV-2 circulation within countries, as suggested by large differences in subnational notification rates [9,10]. From an epidemiological perspective, the imported classification may be more relevant for instances where a tourist introduced the virus when visiting an area in their country of residence than for a case in a worker commuting daily across a European border. In addition, it is not clear whether cases infected in the EU outermost regions or Overseas Countries and Territories should be reported as imported. If the place of residence and probable place of infection are reported at a subnational level, this difficulty may be overcome.

Imported cases are usually included when calculating the notification rate, even when there are few autochthonous cases, such as in the case of dengue [11]. This helps to assess the disease burden for the reporting countries and the potential risk for further transmission in regions where a competent vector is present. For COVID-19, imported cases may represent a risk factor for (re)introduction of SARS-CoV-2, especially in a situation where local transmission has been interrupted or reduced to very low levels and mitigation measures have been relaxed. It is thus probably best to include imported cases in the notification rate, even if their role in furthering transmission may be limited. However, as imported cases may also reflect testing strategies that target travellers upon entering a country, the number of imported cases may not reflect the epidemiological situation in said country. Therefore, the incidence may be overestimated when these cases are added to the national total. This may be of concern if these rates are used to inform border closure or quarantine policies for travellers.

Aside from the impact of imported cases on the notification rate, another challenge is to determine the population under surveillance, which is crucial when selecting the denominator used for calculating notification rates. As for many other diseases under comprehensive surveillance, the population under surveillance is the general population. Countries report cases of residents present in the country and of visitors who have fallen ill and been diagnosed during their stay. The inclusion of cases among non-residents could lead to an overestimation of the notification rate if only residents are counted in the denominator. This is generally not problematic, since visitors usually account for only a small proportion of all reported cases. Yet, in countries with a small population, large numbers of tourists or commuters testing positive for the disease under surveillance could impact the overall notification rate substantially, especially if the disease is associated with large outbreaks in specific settings, such as holiday resorts, seasonal worker accommodations or migrant reception centres. Although it may be possible to identify and exclude those diagnosed by entry testing from the numerator (provided that they remain in strict quarantine), most travellers, migrants living in the country or transients would be included. Moreover, people staying for longer periods or with multiple re-entries (e.g. commuters) will be contributing to community transmission regardless of their residency status. For all these reasons, it appears important to include all cases in notification rate calculations. If estimating very precise rates is necessary, adding non-residents to the denominator can be considered for a short period of time (e.g. in the summer months for regions with large numbers of tourists).

The underlying assumption for classifying a case as imported or travel associated is that the probable place of infection can be easily determined, or at least that it is possible to eliminate the reporting country as the probable place of infection. If testing is done upon entry, one could reasonably assume that the case was indeed imported. The predictive value of the definition for importation is unknown for COVID-19 and depends on the current transmission intensity in both the reporting country and the visited country, as well as the length of stay. Since cases may have travelled in more than one country, the ascertainment of the place of infection may be even more challenging. Without sound epidemiological investigation of imported cases and clusters around them, classification of COVID-19 as imported or not will remain unreliable in many instances. There is also a risk that cases may withhold their travel history because of possible inconvenience, such as quarantine [12].

The impact of the inclusion of imported COVID-19 cases in the calculation of the notification rate will depend greatly on the surveillance system in place. Therefore, it is essential to describe the surveillance system as accurately as possible, using surveillance descriptors [13]. These descriptors should be reviewed periodically to document changes, such as an increase of testing. For COVID-19 cases, the most important descriptors are the population under surveillance and the case detection policy. For the former, it should be indicated whether a specific population group is targeted, such as healthcare workers. For the latter, the specific policies in place should be documented, such as the screening of travellers upon entry to a country. In addition, information on notified cases should include reason for testing (e.g. screening of returning traveller).

The collection, analysis and sharing of comprehensive data at national and international levels is essential to allow for appropriate, evidence-based public health action. Particularly in the context of an evolving pandemic, detailed information may have important implications for response. The choice of surveillance indicators, such as the notification rate, should be determined by the surveillance objectives. The inclusion or exclusion of imported cases in these indicators will depend on the objectives. It is of utmost importance that countries report all cases diagnosed in their territories, regardless of their residence or importation status. The inclusion of imported cases should not provide disincentives to test or report and surveillance data should be interpreted by taking into account surveillance system descriptors.
